# Better Together? Coupling Pharmacotherapies and Cognitive Interventions With Non-invasive Brain Stimulation for the Treatment of Addictive Disorders

**DOI:** 10.3389/fnins.2019.01385

**Published:** 2020-01-10

**Authors:** Primavera A. Spagnolo, Chiara Montemitro, Mauro Pettorruso, Giovanni Martinotti, Massimo Di Giannantonio

**Affiliations:** ^1^Human Motor Control Section, Medical Neurology Branch, National Institute on Neurological Disorders and Stroke, National Institutes of Health, Bethesda, MD, United States; ^2^Department of Neuroscience, Imaging and Clinical Sciences, G. D'Annunzio University, Chieti, Italy; ^3^Neuroimaging Research Branch, National Institute on Drug Abuse, Intramural Research Program, National Institutes of Health, Baltimore, MD, United States

**Keywords:** NIBS, addictive disorders, multimodal interventions, combination strategies, neurocircuits

## Introduction

Addictive disorders (AD) are one of the leading causes of morbidity and mortality worldwide (World Health Organization, 2018). Although several pharmacological and behavioral treatments for these disorders have shown efficacy in controlled clinical trials, there is a need for more effective treatments. Recently, there has been an emerging emphasis in investigating neurocircuitry-based treatment options for patients with AD (Diana et al., 2017; Spagnolo and Goldman, 2017). Specifically, an increasing number of studies has evaluated the therapeutic potential of non-invasive bran stimulation (NIBS) techniques, such as repetitive transcranial magnetic stimulation (rTMS) and transcranial direct current stimulation (tDCS), in various substance-dependent populations, as well as in subjects with behavioral addictions. The interest in NIBS has been hastened by advances in the neuroscience of addictive disorders, indicating that neurocircuitry dysfunctions (e.g., cortico-striatal and cortico-limbic circuits) underlie the behavioral and clinical alterations commonly observed in patients with AD (Volkow et al., 2016). Since none of the therapies for AD currently available can undo these neuroadaptations, the possibility to target and restore them via NIBS appears particularly promising.

The use of NIBS for AD, however, is still in its infancy, and several questions, including the optimal target, stimulation protocol, and treatment duration, still need an answer before these interventions could be used as a tool for clinical practice in addiction medicine. In this regard, recent rTMS and tDCS trials in AD patients have contributed to identify several factors playing an important role for NIBS efficacy, such as coil and electrodes orientation, scalp-brain distance, and gray/white matter structure, density and integrity. In addition to those, a further factor that appears to critically modulate the effects of NIBS is the *state of brain* during the application of the stimulation. It is well-known that pharmacotherapies, psychotherapies (e.g., cognitive behavioral therapy, CBT) and behavioral or cognitive tasks affect brain activity and connectivity. Thus, using them in conjunction with neuromodulation interventions may ultimately change treatment outcomes, and also explain the interindividual variability often observed in response to NIBS (Silvanto and Pascual-Leone, 2008; Luber and Lisanby, 2014; Romei et al., 2016).

## State-Dependent Effects of NIBS: The Role of Pharmacotherapies

The “state-dependent” effects of NIBS have been initially studied in regard to pharmacotherapies, and particularly active drugs on the central nervous system (CNS), since these medications have been shown to alter excitability measures and NIBS-induced plasticity (Ziemann et al., 2015; Martinotti et al., 2019). Among the pharmacological interventions evaluated to date, medications such as dextromethorphan (Nitsche et al., 2003), diazepam (Ziemann et al., 2015), baclofen (McDonnell et al., 2007), and propranolol (Nitsche et al., 2004a) appear to block the facilitation or inhibition associated with brain stimulation. However, D-cycloserine (Nitsche et al., 2004b), amphetamine (Nitsche et al., 2004a), and nicotine (Thirugnanasambandam et al., 2011) have been shown to increase the long-term potentiation-like effects of NIBS in healthy individuals, thus suggesting that combining NIBS with pharmacotherapies may also lead to supraordinal effects on neuroplasticity (for a review, see Ziemann et al., 2015).

Few studies have considered this phenomenon also in the context of several psychiatric disorders. For example, a recent observational study in patients with Major Depressive Disorder (MDD) reported that combining rTMS with psychostimulants (e.g., modafinil, methylphenidate) was associated with greater clinical outcomes, compared to other medications (Hunter et al., 2019). Furthermore, atomoxetine combined with rTMS has showed significant clinical advantages compared with both rTMS and atomoxetine in monotherapy (Cao et al., 2018), as well as clozapine efficacy is improved when combined with neuromodulation techniques in clozapine-resistant schizophrenic patients (Arumugham et al., 2016). However, combing deep TMS with SSRIs in patients with treatment-resistant depression was not associated with improved clinical outcomes, compared to deep TMS alone (Tendler et al., 2018). There are presently no studies in the behavioral or substance addiction literature that have directly evaluated the combined effects of NIBS and pharmacotherapy, although several trials have enrolled patients with AD receiving pharmacological treatment (Klauss et al., 2014; Mishra et al., 2015; Del Felice et al., 2016; Wang et al., 2016). Thus, future research should investigate whether concurrent administration of pharmacotherapies could help optimize NIBS therapy, and define the mechanisms by which different medications used for AD interact with NIBS.

## Combining Cognitive Training/Therapy with NIBS

In addition to pharmacological treatments, cognitive and/or behavioral interventions also interact with NIBS, by modulating ongoing neural activity in the targeted circuits and associated networks. The effects of this interaction critically depend on the timing of delivery, as cognitive and behavioral interventions can be applied simultaneously or sequentially to NIBS. Several studies have shown that the behavioral effects of brain stimulation (facilitatory vs. inhibitory) change when TMS is preceded by an initial psychophysical manipulation (Silvanto and Pascual-Leone, 2008; Silvanto et al., 2017). This because brain state manipulations may act as a functional priming of a certain neurocircuitry, which, consequently, may respond differently to neurostimulation (Silvanto et al., 2017). Furthermore, functional engagement of a neurocircuitry with a cognitive task has been proposed to facilitate the long-term potentiation like-effects induced by NIBS (Luber et al., 2007; Tsagaris et al., 2016). Otherwise, when NIBS is applied before or simultaneously to a cognitive or behavioral intervention, it may enhance and facilitate inherent learning processes associated with these interventions, considering the ability of NIBS in boosting DA signaling and the evidences suggesting that strengthening the DA signal may improve memory formation, as well as emotionally relevant information encoding (Cannizzaro et al., 2019). Indeed, TMS has been used in conjunction with cognitive strategies such as CBT or emotional recall in patients with MDD and PTSD, with promising results (Isserles et al., 2011; Neacsiu et al., 2018).

However, the temporal relationship between multimodal interventions remains relatively an unexplored territory (Tsagaris et al., 2016), and is one of the most poorly reported variables in NIBS studies, although identifying the optimal timing of combined interventions may enhance their therapeutic effects, while also helping to avoid inducing maladaptive plasticity.

With regard to the combined effects of NIBS and psychotherapy, research has mainly focused on patients with mood and anxiety disorders, with mixed results (for a review, see Chalah and Ayache, 2019). Differences in the type of psychotherapy as well as in the number of sessions may explain the inconsistency among studies, although cognitive-behavioral therapy seems to enhance the top-down modulatory effects of prefrontal stimulation (Tan et al., 2015; Grassi et al., 2018). In the field of addictive disorders, preliminary evidence suggests that NIBS is most likely to be effective when combined with evidence-based self-help intervention or cognitive-behavioral interventions, as indicated by several studies evaluating the effects of TMS for nicotine addiction (for a review, see Hauer et al., 2019).

One of the most interesting areas of recent methods development in NIBS involves choosing a task for the participants to perform before or during the stimulation. Dinur-Klein et al. (2014) were the first to demonstrate that it is possible to amplify the effects of TMS on smoking cessation by having individuals engage in a smoking cue-reactivity task immediately before the TMS session (Dinur-Klein et al., 2014). Specifically, in this large, double-blind, sham-controlled study of 115 cigarette smokers, half of the participants were presented with visual and olfactory smoking cues before the TMS session (deep TMS targeting insula and lateral prefrontal cortex bilaterally). Individuals that had received high-frequency deep TMS in conjunction to smoking cues exposure exhibited significantly lower cigarette consumption and nicotine dependence than sham TMS. Similar results have also been observed in Obsessive Compulsive Disorder (OCD) patients receiving high-frequency deep TMS of the medial prefrontal cortex (mPFC)—anterior cingulate cortex (ACC) region following exposure to individualized, obsessive-compulsive cues (Carmi et al., 2018). These results suggest that task-induced plasticity may enhance the behavioral effects of rTMS, although the precise mechanism mediating this phenomenon has not been directly investigated in patients with AD or with other psychiatric conditions.

In addition to cue exposure paradigms, which engage brain circuits mediating cue reactivity, for NIBS studies targeting prefrontal control circuitry [i.e., the dorsolateral prefrontal cortex—DLPFC, a major node of the executive control network (ECN)] the choice of a cognitive task may be the best approach to maximize the benefits to be gained from either intervention. Supporting this concept, emerging evidence indicates that simultaneous tDCS and cognitive control therapy (CCT), a neurocognitive intervention for MDD that engages the left DLPFC (Brunoni et al., 2014), has stronger antidepressant effects compared to tDCS alone (Brunoni et al., 2014; Segrave et al., 2014). Interestingly, the antidepressant effect positively correlated with cognitive performances during CCT, thus suggesting that enhanced cognitive control via tDCS + CCT mediated the clinical outcomes (Vanderhasselt et al., 2015). Similarly, addition of tDCS to working memory tasks has been shown to enhance long-term cognition in schizophrenics (Orlov et al., 2017), while combining tDCS with an attentional bias modification task reduced reactivity to negative environmental stimuli in anxious individuals (Heeren et al., 2017).

Preliminary evidence in the field of AD have also been reported. Specifically, in a recent trial in patients with alcohol use disorders (AUD), 4 sessions of attentional bias training (control or real) were combined with either sham or active tDCS over the DLPFC, using a 2-by-2 double-blind factorial design (den Uyl et al., 2018). Combined active tDCS and real training did not produce any significant effect on alcohol craving and relapse, and on attentional biases toward alcohol. However, as also observed by the authors, individuals enrolled in the study had low baseline craving levels. Furthermore, the number of sessions delivered may not have been enough to produce a clinical meaningful effect (Spagnolo and Goldman, 2017). Interestingly, a further study found that tDCS over the left DLPFC significantly decreased the engagement bias toward drug cues in abstinent methamphetamine users (Shahbabaie et al., 2018). Finally, a recent study evaluated the effects of 4 sessions of combined tDCS targeting the right inferior frontal gyrus and cognitive bias modification training in high-risk drinkers (AUDIT score >8) and found no effect on drinking measures or alcohol approach biases (Claus et al., 2019).

## Discussion

The behavioral and clinical effects of NIBS depends on what the brain is doing at the time of stimulation. Brain state can be affected by pharmacotherapies, as well as by behavioral and cognitive interventions, which act by modulating and/or engaging disease-related circuits targeted via neurostimulation. Increasing evidence suggest that this combined approach can be useful for treating various psychiatric disorders (for a review, see Sathappan et al., 2019), and could prove to be a promising approach worth further examination also in the field of AD. Indeed, multimodal, integrated interventions are successfully used to treat patients with chronic conditions.

However, several important issues should be investigated to fully delineate the therapeutic potentials of combined therapies for AD. In particular, attention should be devoted to the complex interplay between AD and factors known to modulate response to both NIBS and cognitive interventions. For example, prolonged exposure to addictive agents has been shown to impair cortical plasticity, including motor cortical plasticity (Huang et al., 2017; Shen et al., 2017), an effect which can reduce response to NIBS protocols. Neurostimulation effects on brain plasticity can also be affected by genetic factors, including polymorphisms at the level of the Brain-Derived Neurotrophic Factor (BDNF) gene (Cheeran et al., 2008). Importantly, many addictive agents lead to changes in endogenous BDNF expression in neural circuits implicated in AD (Barker et al., 2015), thus indicating as response to NIBS is modulated by a complex interaction between stimulation-related factors, individual factors, and AD-related factors. A further example is represented by sex-differences and endogenous estrogen levels, which have been associated to variability in response to both TMS and cognitive interventions (Glover et al., 2015; Chung et al., 2019), and with changes in BDNF levels (Barker et al., 2015).

Taken together, these observations strongly support the need to better characterize the biobehavioral responses to both neuromodulation (TMS, tDCS) and other interventions (cognitive bias modification, medications, psychotherapy). For NIBS, this requires addressing questions related to stimulation parameters, brain targets, number of sessions, factors influencing the stimulation dose delivered, and tools to measures the neurophysiological, circuit-level and behavioral effects of neuromodulation interventions.

With regard to pharmacotherapies, while their action on cortical excitability and brain plasticity have been studies, it will be also critical to define how medications currently used for AD modulate brain activity and connectivity. For example, naltrexone, a medication commonly used in patients with alcohol and opioid use disorders, has been shown to modulate brain connectivity (Morris et al., 2018; Elton et al., 2019). Since NIBS also has a modulatory effect on brain connectivity, particularly when applied to network nodes (Eldaief et al., 2011), future research should investigate whether these effects can be combined in a synergistic fashion.

For psychotherapies, quantifying dose is more challenging since both number and duration of treatment sessions should be evaluated, and optimal measures of treatment responses, which take in consideration the specificity of this interventions (e.g., therapeutic relationship between patient and therapist, internal state of the patient during time of therapy), are still missing. Furthermore, as for medications, the documented effects of CBT on brain connectivity should be studied in the context of combined therapy with NIBS. Mason et al. (2015) reported that CBT increased DLPFC connectivity with amygdala in patients with psychosis, an effect which predicted subsequent recovery (Mason et al., 2015). This may suggest that coupling this intervention with NIBS targeting the prefrontal control circuit may enhance CBT effects on corticolimbic connectivity.

With regard to behavioral and cognitive tasks, several critical factors should be considered when evaluating the effects of these intervention both alone and in combination with NIBS. With regard to cue exposure paradigms, a recent study has indicated that individual's baseline frontal-striatal reactivity to cues modulates the effects of TMS targeting the medial PFC. This underscores the importance of assessing individual variability with the aim to identify subjects who can benefit more from these interventions (Kearney-Ramos et al., 2019). Similarly, cognitive bias modification efficacy varies whether it is tested in problematic drinkers vs. treatment-seeking patients with AD (Wiers et al., 2018). This is not surprising, as expectations -of a drug or of a clinical benefit -modulate brain responses and affect outcomes (Spagnolo et al., 2015).

As the field continues to grow, we are optimistic the future studies will be designed to address these questions, and that significantly more attention will be given to combined therapies, with the hope to provide a novel, tailored and effective treatment approach to patients with AD.

## Author Contributions

PS and CM designed the paper and reviewed the literature. PS, CM, MP, GM, and MD wrote the opinion paper and reviewed the manuscript. All authors approved the final paper.

### Conflict of Interest

The authors declare that the research was conducted in the absence of any commercial or financial relationships that could be construed as a potential conflict of interest.
